# Early Mortality Was Highly and Strongly Associated with Functional Status in Incident Japanese Hemodialysis Patients: A Cohort Study of the Large National Dialysis Registry

**DOI:** 10.1371/journal.pone.0156951

**Published:** 2016-06-07

**Authors:** Masahiko Yazawa, Ryo Kido, Seiji Ohira, Takeshi Hasegawa, Norio Hanafusa, Kunitoshi Iseki, Yoshiharu Tsubakihara, Yugo Shibagaki

**Affiliations:** 1 Division of Nephrology and Hypertension, St Marianna University School of Medicine, Kawasaki, Kanagawa, Japan; 2 Institute for Health Outcomes and Process Evaluation Research (iHope International), Kyoto, Japan; 3 Sapporo Kita Clinic, Sapporo, Hokkaido, Japan; 4 Center for Innovative Research for Communities and Clinical Excellence, Fukushima Medical University, Fukushima, Japan; 5 Division of Total Renal Care Medicine, The University of Tokyo Hospital, Tokyo, Japan; 6 The Committee of Renal Data Registry, Japanese Society for Dialysis Therapy, Tokyo, Japan; Nagoya University, JAPAN

## Abstract

**Background:**

Although dialysis is typically started in an effort to prolong survival, mortality is reportedly high in the first few months. However, it remains unclear whether this is true in Japanese patients who tend to have a better prognosis than other ethnicities, and if health conditions such as functional status (FS) at initiation of dialysis influence prognosis.

**Methods:**

We investigated the epidemiology of early death and its association with FS using Japanese national registry data in 2007, which included 35,415 patients on incident dialysis and 7,664 with FS data. The main outcome was early death, defined as death within 3 months after initiation of hemodialysis (HD). The main predictor was FS at initiation of HD. Levels of functional disability were categorized as follows: severe (bedridden), moderate (overt difficulties in exerting basic activities of daily living), or mild/none (none or some functional disabilities).

**Results:**

Early death remained relatively common, especially among elderly patients (overall: 7.1%; those aged ≥80 years: 15.8%). Severely and even only a moderately impaired FS were significantly associated with early death after starting dialysis (adjusted risk ratios: 3.93 and 2.38, respectively). The incidence of early death in those with impaired FS increased with age (36.5% in those with severely impaired FS and aged ≥80 years).

**Conclusions:**

Early death after starting dialysis was relatively common, especially among the elderly, even in Japanese patients. Further, early death was significantly associated with impaired FS at initiation of HD.

## Introduction

Dialysis is initiated in patients with end-stage kidney disease (ESKD) primarily to prolong survival; however, mortality in the first few months after starting dialysis is reportedly higher than later mortality [1]. A recent analysis in Western countries found that the probability of early death (within 3 months after initiation of dialysis) ranged from 5.6 to 8.6% [2], indicating that many patients do not survive long enough to benefit from dialysis. Early death after initiating dialysis is therefore a relevant problem that should be recognized before renal replacement therapy becomes necessary and should be discussed with patients and families. The few studies that have addressed this issue have been limited to Western populations [1, 3–6]; thus, it is unknown whether this is also true in Japanese maintenance hemodialysis patients who have a better long-term prognosis.

The prognosis of patients with ESKD following initiation of dialysis has been suggested to be related to the health conditions present at the time of dialysis initiation [6–8]. Activities of daily living (ADL) may be one of the most important prognostic factors, given the dramatic increase in the proportion of frail elderly patients on dialysis [9, 10]. Although dialysis is initiated in this particular subpopulation in an effort to reverse frailty with amelioration of uremia, it can worsen performance of ADL [11] and consequently patient prognosis [12, 13]. In Japan, this is a particularly relevant issue, as no guidelines have been developed for withholding/withdrawing dialysis, and doctors are not exempt from lawsuits for not initiating life-prolonging treatment. Dialysis is therefore initiated in most patients with ESKD irrespective of their functional status (FS) as long as they can tolerate the procedure. The scoring system used in Japan to evaluate indications for dialysis assigns a higher score to patients with more severe functional disabilities [14].

We evaluated the epidemiology of early death after initiation of dialysis using Japanese national registry data, as well as the relationship between FS and the incidence of early death after initiation of incident hemodialysis (HD) in adult Japanese patients.

## Materials and Methods

### Study design and subjects

The present observational study used national dialysis registry data, and permission was obtained from the Statistical Committee of Renal Data Registry of the Japanese Society for Dialysis Therapy. We used the standard analysis file (JRDR-09005), which contained patient data gathered annually from >99% of all HD facilities in Japan. The study protocol was approved by the Medicine Ethics Committee of the Japanese Society for Dialysis Therapy.

All 35,415 patients who initiated incident HD in 2007 were enrolled using the following exclusion criteria: those aged <20 years (n = 106), those withdrawn from HD within 3 months of initiation (n = 211), those who underwent kidney transplantation (n = 67), and those who lacked any basic demographic data (e.g., age, sex, and outcome of survival; n = 1,750). We then determined the incidence of death within 1 year of initiation of HD and early death, defined as death within 3 months of initiation. Next, we analyzed the relationship between FS and early death in the 7,664 patients for whom FS at the time of initiation of HD had been recorded.

The data contained no identifying personal information. The study was conducted in accordance with Japan’s privacy protection laws and ethical guidelines for epidemiological studies published by the Ministry of Education, Science and Culture, and the Ministry of Health, Labor, and Welfare in 2005, and the STROBE guideline.

### Outcomes, predictors, and covariates

The main outcome was early death defined as death within 3 months of initiation of HD. The secondary outcome was the cumulative incidence of all-cause deaths within 6 and 12 months.

Primary causes of death were categorized as follows: vascular disease (defined as ischemic heart disease, congestive heart failure, valvular disease, cardiomyopathy, pericarditis, myocarditis, arrhythmia, subarachnoid hemorrhage, intracerebral hemorrhage, another type of cerebrovascular disease, and sudden death), infection (defined as sepsis, tuberculosis, HIV, pneumonia, influenza, and other), cancer, and other.

The main predictor was FS at initiation of HD. Levels of FS in patients with ESKD and uremia were categorized as follows: severe (bedridden), moderate (overt difficulties in exerting basic ADL), or mild/none (none or some functional disabilities that are not moderate or severe). These categories have been defined as the inability to perform daily activities (activities of daily life) in the Japanese guideline for initiating dialysis [14].

FS is one of three items outlined in the scoring system and described in the Guideline for Initiating Dialysis, which has been used in clinical situations for the last two decades in Japan [14]. Scores were assigned based on summed sub-scores from three categories: clinical symptoms, kidney function (serum creatinine levels or creatinine clearance), and FS. However, these categories are not evaluated equally, and patients with more severe FS tend to receive higher scores; indeed, 90% of Japanese physicians have admitted to placing greater importance on FS, which influences initiation of HD [15]. FS in this case is evaluated by nephrologists or dialysis physicians based on patients’ clinical course and subjective health condition, without referencing laboratory findings or determining ADL using validated methods.

Covariates were fixed baseline parameters at initiation of HD, with age, sex, and the cause of ESKD as case-mix variables, and the following variables were used for multivariable analysis: body mass index, systolic blood pressure, comorbid conditions (i.e., congestive heart failure, ischemic heart disease, stroke, diabetes mellitus, malignancy, dementia, and liver disease), history of amputation, treatment time of dialysis, type of vascular access, late referral to a nephrologist (defined as ≤90 days from the first visit to initiation of dialysis) [16], and laboratory findings (the estimated glomerular filtration rate [eGFR] calculated from the formula for Japanese: 194 × [serum creatinine, mg/dL]^−1.094^ × [age, years]^−0.287^, and × 0.739 if female [17]; and levels of albumin, hemoglobin, C-reactive protein [CRP], calcium [albumin-adjusted: calcium + 4.0 –albumin, if the albumin level is <4.0 g/dL] [18], and phosphorus).

### Statistical analysis

To determine the difference in distribution among the levels of functional disability, we used the Kruskal-Wallis and χ^2^ tests to determine baseline characteristics of continuous variables (median [interquartile range; IQR]) and categorical variables (%), respectively. FS and the incidence of early death in each age group were documented graphically.

In our main analysis, we computed risk ratios and 95% confidence intervals (CIs) for early mortality among patient groups with different levels of functional disability at initiation of HD. To adjust for the distribution of covariates among those groups, we used modified Poisson regression with robust variance [19]. Considering the potential for misclassifying FS, especially between mild/none and moderate, we conducted a sensitivity analysis between the severe and non-severe groups (mild/none + moderate).

To analyze the relationship between FS and early death with regard to age, we used four age categories (<60, 60–69, 70–79, and ≥80 years) with three FS levels (mild/none, moderate, and severe) to obtain 12 categories for use as variables. Finally, we used multivariable regression analysis to determine the relationship between FS and early death according to each age category. The data sets used to analyze the relationship between FS and early death (n = 7,664) had several missing values (Table 1). For covariate data missing at baseline, we used multiple imputation by IVEware (University of Michigan; http://www.isr.umich.edu/src/smp/ive/) to account for this uncertainty. Missing data were sequentially imputed based on multiple regression models with other variables as covariates, according to the type of variable missing. Each imputed data set was then constructed by repeating this sequential procedure for 10 iterations. Results were obtained using Rubin’s formula with five imputed data sets [20]. P-values <0.05 were considered statistically significant. Statistical analysis was performed with SAS, version 9.2 (SAS Institute, Cary, NC, USA).

**Table 1:** Distribution of Early Mortality After the Initiation of Hemodialysis and Primary Causes of Death in Japan (n = 33,281).

			Primary Cause of Death			
	Number of deaths/Number of patients, n (%)		*Cancer*, *n (%)*	*Vascular disease*, *n (%)*	*Infection*, *n (%)*	*Others*, *n (%)*
*Death within 3 months*						
Age, years						
<60	212/9,076	(2.3%)	34 (16.0%)	70 (33.0%)	44 (20.8%)	64 (30.2%)
60 to 69	396/8,379	(4.7%)	54 (13.6%)	127 (32.1%)	99 (25.0%)	116 (29.3%)
70 to 79	876/10,308	(8.5%)	85 (9.7%)	302 (34.5%)	227 (25.9%)	262 (29.9%)
≥80	874/5,518	(15.8%)	54 (6.2%)	329 (37.6%)	210 (24.0%)	281 (32.2%)
Total	2,358/33,281	(7.1%)	227 (9.6%)	828 (35.1%)	580 (24.6%)	723 (30.7%)
*Death within 12 months*†						
Age, years						
<60	466/8,948	(5.2%)	70 (15.0%)	167 (35.8%)	81 (17.4%)	148 (31.8%)
60 to 69	819/8,350	(9.8%)	121 (14.8%)	260 (31.8%)	181 (22.1%)	257 (31.4%)
70 to 79	1,830/10,273	(17.8%)	214 (11.7%)	647 (35.4%)	439 (24.0%)	529 (28.9%)
≥80	1,658/5,494	(30.2%)	124 (7.5%)	629 (37.9%)	368 (22.2%)	537 (32.4%)
Total	4,773/33,065	(14.4%)	529 (11.1%)	1,703 (35.7%)	1,069 (22.4%)	1,471 (30.8%)

*Note*: Values for categorical variables are given as numbers (percentage).

^†^: Patients who stopped receiving hemodialysis 3 months or more after the initiation of hemodialysis were excluded. During the 3 to 6 months after starting hemodialysis, 105 patients terminated hemodialysis and 65 received transplantation; during the 6 to 12 months after starting hemodialysis, 38 patients terminated hemodialysis and 73 received transplantation.

## Results

### Early deaths among Japanese patients on incident HD

**Fig 1:**
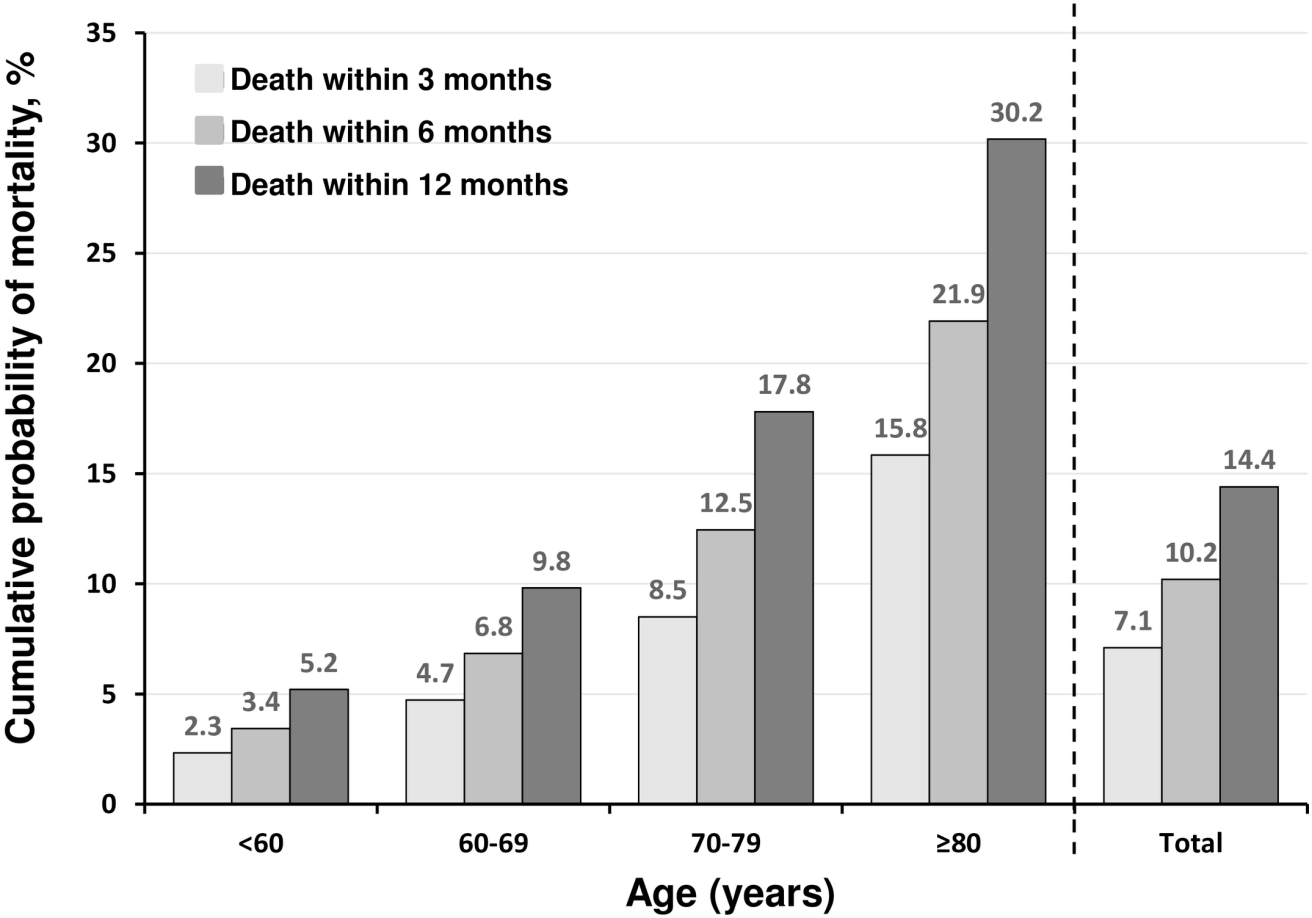
Cumulative probability of early mortality 3, 6, and 12 months after the start of hemodialysis by age (n = 33,281). Patients who stopped receiving hemodialysis 3 months or more after the initiation of hemodialysis were excluded. During the 3 to 6 months after starting hemodialysis, 105 patients terminated hemodialysis and 65 received transplantation; during the 6 to 12 months after starting hemodialysis, 38 patients terminated hemodialysis and 73 received transplantation.

The median age of initiation of HD was 69 years (IQR, 59–77 years), and the prevalence of incident HD among women was 35%. The incidence of early deaths was 2,358 (7.1%) of 33,281 eligible patients; 10.2% and 14.4% died within 6 and 12 months, respectively (Fig 1). The incidence of early death tended to be higher among older patients, especially those aged ≥80 years, with incidence of death within 3 and 12 months 15.8% and 30.2%, respectively. Thus, early death accounted for about one-half of all deaths within 12 months in every age group (overall, 7.1% of 14.4%; aged ≥80 years, 15.8% of 30.2%) (Fig 1).

The main causes of death are shown in Table 1. Vascular diseases (35.1%) and infection (24.6%) accounted for almost 60% of early deaths. The proportion of vascular diseases as the cause of early death increased slightly as patients aged, whereas the proportion of cancer as the cause of early deaths decreased with patient age. Infection was responsible for as many as 25% or slightly less of early deaths in all age groups. Similar trends were noted for deaths within 6 and 12 months.

### Characteristics of patients according to their FS at initiation of HD

Baseline characteristics of 7,664 patients with data available on FS at initiation of HD are shown in Table 2. The median age was 69 years (IQR, 59–77 years), and women accounted for 36.1% of subjects. Diabetic kidney disease was the most common cause of ESKD (48.1%) followed by chronic glomerulonephritis (CGN; 26.6%), and prevalent co-morbidities were diabetes mellitus (50.7%), congestive heart failure (28.6%), and stroke (19.7%). About one-third of patients used temporary catheters at initiation of HD, and the mean eGFR was 4.8 mL/min/1.73 m^2^. In addition, we analyzed differences among those with three levels of functional disability using *post-hoc* multiple comparisons with Bonferroni procedure in baseline characteristics (S1 Table).

**Table 2:** Baseline Characteristics of Subjects Compared Among Those with Different Functional Status (n = 7,664).

	Total		Levels of Functional Disability			
Variables	*Value*	*n*	*Mild/None (n = 3*,*192)*	*Moderate (n = 2*,*935)*	*Severe (n = 1*,*537)*	p-value*
*Demographic and clinical characteristics*						
Age, years	69 (59–77)	7,664	66 (57–75)	71 (61–78)	71 (62–79)	<0.001
Sex, female, %	36.1	7,664	32.3	39.2	38.1	<0.001
Body mass index, kg/m^2^	22.5 (20.3–25.2)	5,489	22.7 (20.4–25.2)	22.6 (20.2–25.3)	22.3 (19.8–25.1)	0.006
Cause of end-stage kidney disease		7,024				<0.001
Chronic glomerulonephritis, %	26.6	-	30.7	24.7	21.5	-
Diabetic nephropathy, %	48.1	-	46.8	50.2	47.2	-
Glomerulosclerosis, %	13.3	-	12.0	14.0	14.4	-
Rapid progressive glomerulonephritis, %	1.8	-	1.1	1.8	3.6	-
Others, %	10.2	-	9.5	9.5	13.4	-
Systolic blood pressure, mmHg	153 (136 to 170)	6,828	154 (138–170)	154 (136–172)	150 (130–169)	<0.001
*Co-morbid conditions*						
Congestive heart failure, %	28.6	7,391	18.8	32.8	40.6	<0.001
Ischemic heart disease, %	9.7	7,570	7.6	11.0	12.1	<0.001
Stroke, %	19.7	7,588	14.3	23.4	23.8	<0.001
Diabetes mellitus, %	50.7	7,568	48.6	53.2	50.4	0.002
Malignancy, %	7.1	7,550	5.3	8.7	7.9	<0.001
Hemiplegia, %	6.2	7,516	3.0	7.8	9.5	<0.001
Dementia, %	9.5	7,489	4.0	12.1	16.0	<0.001
Liver disease, %	7.2	7,516	5.9	8.3	7.6	0.001
Past history of amputation, %	1.8	6,890	1.4	2.3	1.9	0.046
*Dialysis*						
Late referral to nephrologist, %†	51.5	6,949	48.9	51.5	57.3	<0.001
Type of vascular access		7,430				<0.001
Arteriovenous fistula, %	51.4	-	61.9	48.9	34.4	-
Temporary catheter, %	33.9	-	22.2	37.4	51.7	-
Others, %	14.7	-	15.9	13.7	13.9	-
Treatment time, hours	3.5 (3.0–4.0)	6,153	3.5 (3.0–4.0)	3.5 (3.0–4.0)	3.02 (3.0–4.0)	0.02
*Laboratory data*						
Albumin, g/dL	3.3 (2.8–3.7)	6,797	3.4 (3.0–3.8)	3.2 (2.8–3.6)	3.1 (2.6–3.5)	<0.001
Hemoglobin, g/dL	8.3 (7.3–9.4)	7,278	8.4 (7.4–9.5)	8.2 (7.3–9.3)	8.3 (7.3–9.4)	<0.001
Estimated glomerular filtration rate, ml/min/1.73 m^2^§	4.8 (3.7 to 6.4)	6,583	4.63 (3.59–6.00)	4.85 (3.68–6.62)	4.91 (3.78–6.89)	<0.001
C-reaction protein, mg/dL	0.36 (0.10–1.96)	5,920	0.20 (0.08–0.80)	0.44 (0.10–2.16)	1.13 (0.20–4.79)	<0.001
Calcium, mg/dL‡	8.7 (8.1–9.2)	6,559	8.6 (8.0–9.1)	8.8 (8.2–9.3)	8.8 (8.3–9.4)	<0.001
Phosphorus, mg/dL	5.6 (4.6–6.8)	6,848	5.6 (4.6–6.8)	5.5 (4.5–6.7)	5.7 (4.5–7.0)	0.02

*Note*: Values for categorical variables are given as numbers (percentage) and continuous variables are given as medians and interquartile ranges (IQR).

^†^: Late referral was defined as 90 days or less from the first day of visit to the initiation of dialysis.

^§^: Estimated glomerular filtration rate was calculated from the formula for Japanese as follows: 194 × [serum creatinine, mg/dL]^−1.094^ × [age, years]^−0.287^, and × 0.739 if female

^‡^: Albumin-adjusted value was used: calcium + 4.0 –albumin (if albumin level <4.0 g/dL). Conversion factors for units: calcium in mg/dl to mmol/L, ×88.4; phosphorus in mg/dl to mmol/L, ×0.3229.

*To test the difference of distribution in for each variable among those within the three different levels of functional disability, we used the χ^2^ tests for categorical variables (%) and the Kruskal-Wallis test for continuous variables.

Regarding FS at initiation of HD, those with significantly impaired FS accounted for more than half of all eligible patients (moderate group, n = 2,935; severe group, n = 1,537; total, n = 7,664). Patients with significantly impaired FS (the moderate or severe group) tended to be older, female, have CGN as a cause of ESKD less often, have more co-morbidities (e.g., vascular diseases, malignancy, liver disease, and dementia), have a higher prevalence of temporary catheter as a type of vascular access, and have lower serum albumin and higher CRP levels, all of which can be judged as clinically significant.

**Fig 2:**
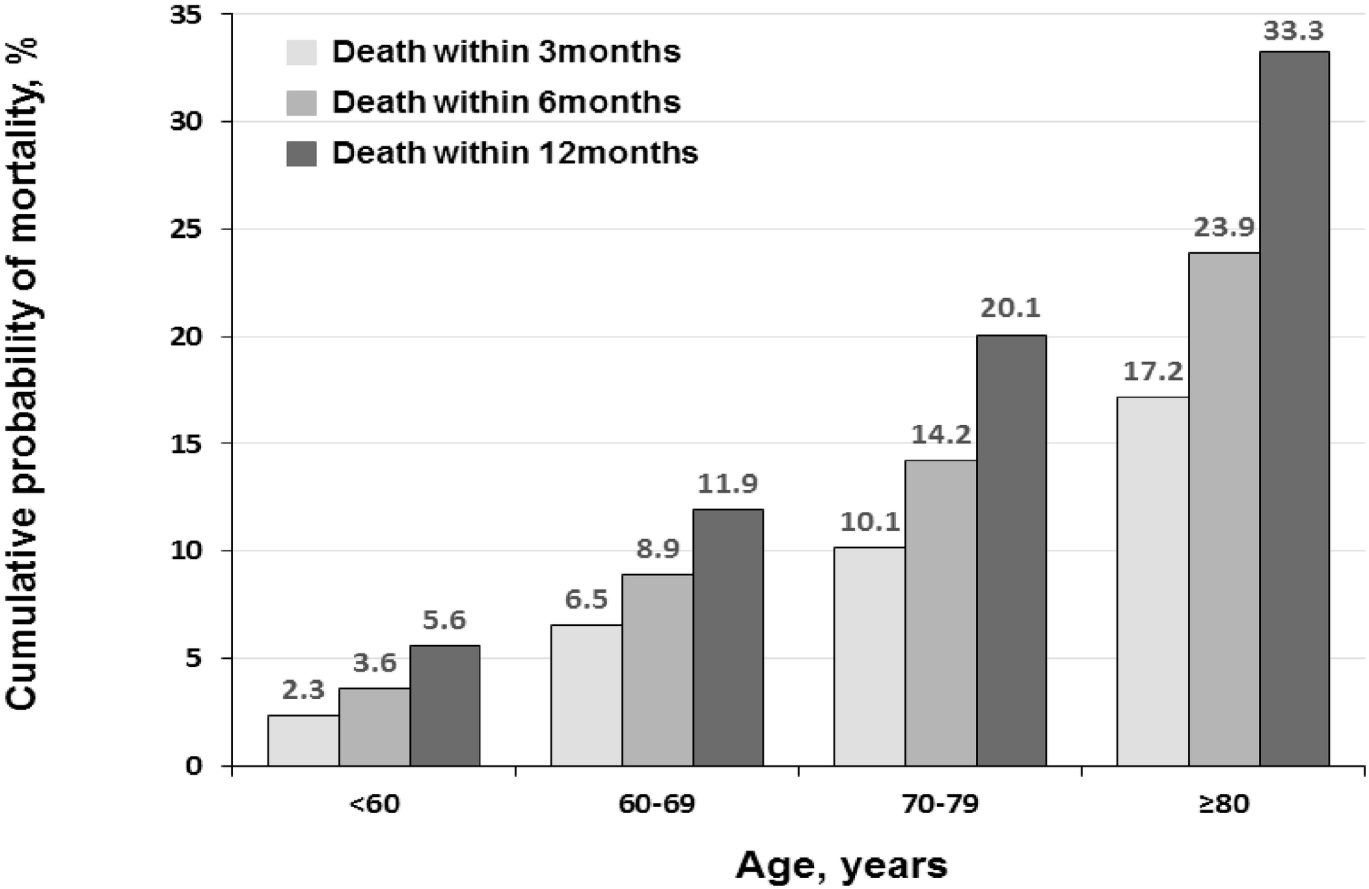
Cumulative Probability of Early Mortality 3, 6, and 12 months After the Start of Hemodialysis by Age (n = 7,664). Patients who stopped receiving hemodialysis 3 months or more after the initiation of hemodialysis were excluded.

The enrolled cohort tended to be older and female, and they died early compared to the excluded cohort (Table 3). However, distribution of the incidence of early death in the enrolled cohort was similar to that among all patients on incident HD (Fig 2).

**Table 3:** Baseline Characteristics and All-cause Mortality Compared between Those Who Were Enrolled and Excluded from This study.

	Total	Study population		
		Enrolled	Excluded	
Variables	n = 33,281	n = 7,664	n = 25,617	p-value*
*Demographic and clinical characteristics*				
Age, years	69 (59 to 77)	69 (59 to 77)	68 (59 to 76)	<0.001
Sex, female, %	35.0	36.1	34.6	0.02
*All-cause mortality early after the initiation of hemodialysis*				
Death within				
3 months, %	7.09	8.44	6.68	<0.001
6 months, %†	10.2	11.8	9.65	<0.001
12 months, %†	14.4	16.6	13.8	<0.001

*Note*: Values for continuous variables are given as medians and interquartile ranges (IQR).

^†^: Patients who stopped receiving hemodialysis 3 months or more after the initiation of hemodialysis were excluded: during the 3 to 6 months after starting hemodialysis, 105 patients terminated hemodialysis and 65 patients received transplantation; during the 6 to 12 months after starting hemodialysis, 38 patients terminated hemodialysis and 73 patients received transplantation.

*To test the difference of distribution in for each variable between enrolled and excluded population, we used the χ^2^ tests for categorical variables (%) and the Mann-Whitney U test for continuous variables.

### Association between FS and early death

The relationship between FS and early death among 7,664 patients with FS data is shown in Table 4. The incidence of early death in the mild/none, moderate, and severe FS groups were 2.0%, 8.1%, and 22.3%, respectively. Crude risk ratios (CI) of early death were 4.00 (95% CI: 1.11–1.60) in the moderate group and 11.0 (95% CI: 8.41–14.3) in the severe group, with the mild/none group as a reference. This trend of higher risk with more severely impaired FS persisted even after adjusting for case-mix variables. Multivariate analysis also showed this trend of increasing risk of early death as FS worsened (moderate, risk ratio: 2.38, 95% CI: 1.8–3.1; severe, risk ratio: 3.93, 95% CI: 2.96–5.22).

**Table 4:** Association of Functional Status with Early Mortality After the Start of Hemodialysis.

Outcomes	Levels of Functional Disability		
	*Mild/None*	*Moderate*	*Severe*
*Death within 3 months*			
Number of events / Number of patients	65/3,192	239/2,935	343/1,537
Proportion, %	2.04	8.14	22.3
Risk ratio of death (95% confidence interval) †			
Univariate analysis	Reference	4.00 (1.11 to 1.60)	11.0 (8.41 to 14.3)
Case-mix adjusted	Reference	3.41 (2.59 to 4.50)	8.39 (6.42 to 11.0)
Multivariate adjusted	Reference	2.38 (1.80 to 3.10)	3.93 (2.96 to 5.22)
*Death within 6 months*			
Number of events / Number of patients	102/3,184	352/2,925	450/1,533
Proportion, %	3.20	12.0	29.4
Risk ratio of death (95% confidence interval) †			
Univariate analysis	Reference	3.76 (3.01 to 4.60)	9.16 (7.39 to 11.4)
Case-mix adjusted	Reference	3.21 (2.57 to 4.01)	7.14 (5.74 to 8.87)
Multivariate adjusted	Reference	2.30 (1.83 to 2.88)	3.47 (2.76 to 4.37)
*Death within 12 months*			
Number of events / Number of patients	199/3,176	530/2,918	538/1,529
Proportion, %	6.27	18.2	35.2
Risk ratio of death (95% confidence interval) †			
Univariate analysis	Reference	2.90 (2.46 to 3.40)	5.62 (4.77 to 6.61)
Case-mix adjusted	Reference	2.48 (2.11 to 2.93)	4.44 (3.76 to 5.24)
Multivariate adjusted	Reference	1.83 (1.54 to 2.16)	2.35 (1.97 to 2.81)

^†^: Modified Poisson regression with robust variance was used to compute the risk ratios and 95% confidence intervals which were adjusted for the difference of the distribution in baseline characteristics as covariates.

To make sure of significant factors other than FS affecting the early death, we revealed multivariable-adjusted risk ratios of each baseline characteristic for early death after initiation of hemodialysis (S2 Table).

FS correlated with early death and death within 6 and 12 months in the severe and moderate groups. Further, sensitivity analysis confirmed significantly high risk ratios of death within 3 months in the severe group compared to the non-severe group (RR 2.06, 95%CI 1.74 to 2.43). This trend is the same as for death within 6 and 12 months.

### Association between other baseline characteristics and early death

Multivariable-adjusted risk ratios of each baseline characteristic for early death after initiation of HD is shown in S1 Table. Age, Sex, rapid progressive glomerulonephritis, temporary catheter use, and hypoalbuminemia were significant risk of early death.

### Association between FS and early death by age category at initiation of HD

**Fig 3:**
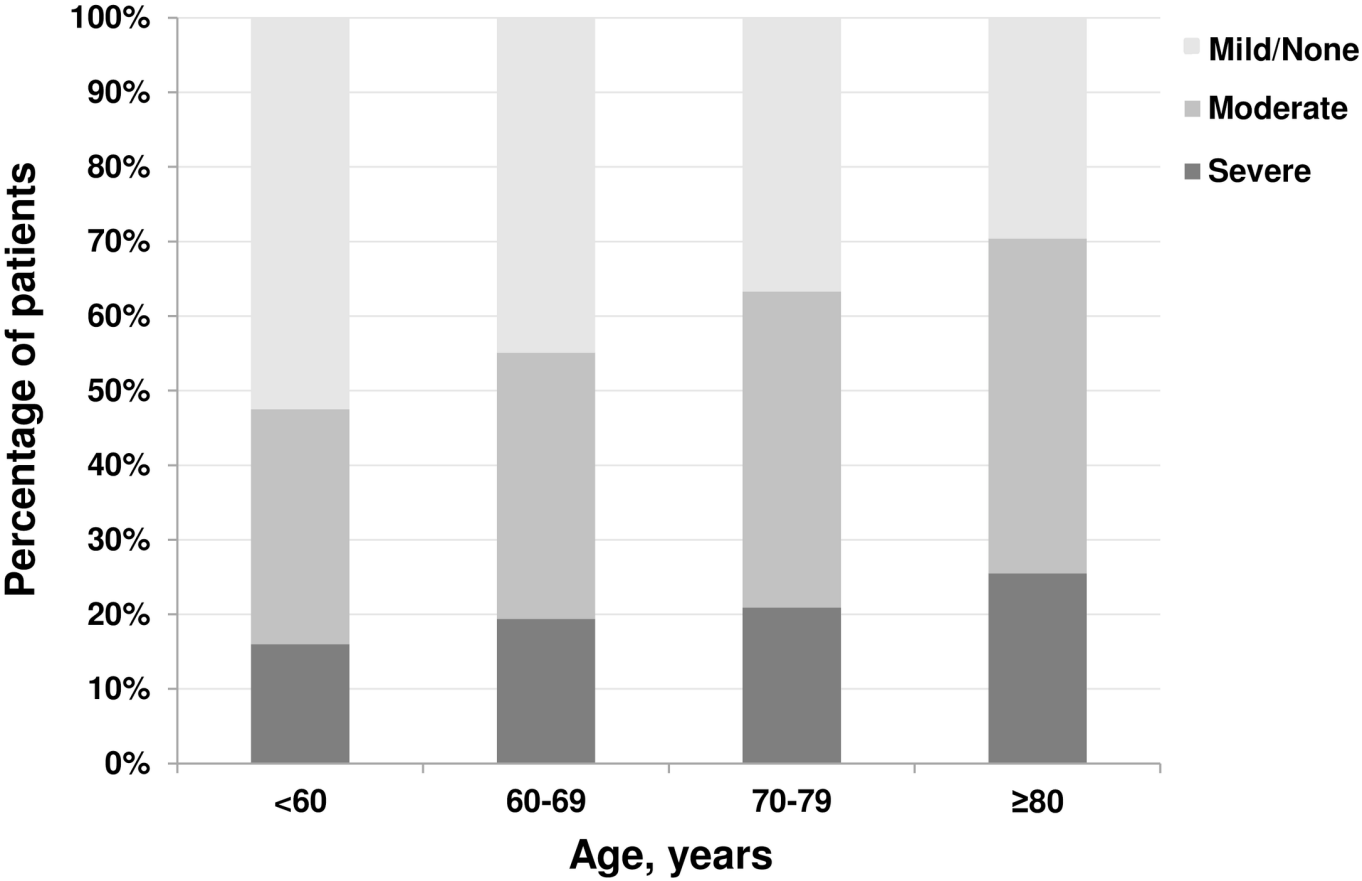
Distribution of functional status of patients at the start of hemodialysis by age (n = 7,664). Disability levels of functional status were categorized in three groups: mild or none, moderate, and severe. Definitions of each category have been described in the Methods section.

**Fig 4:**
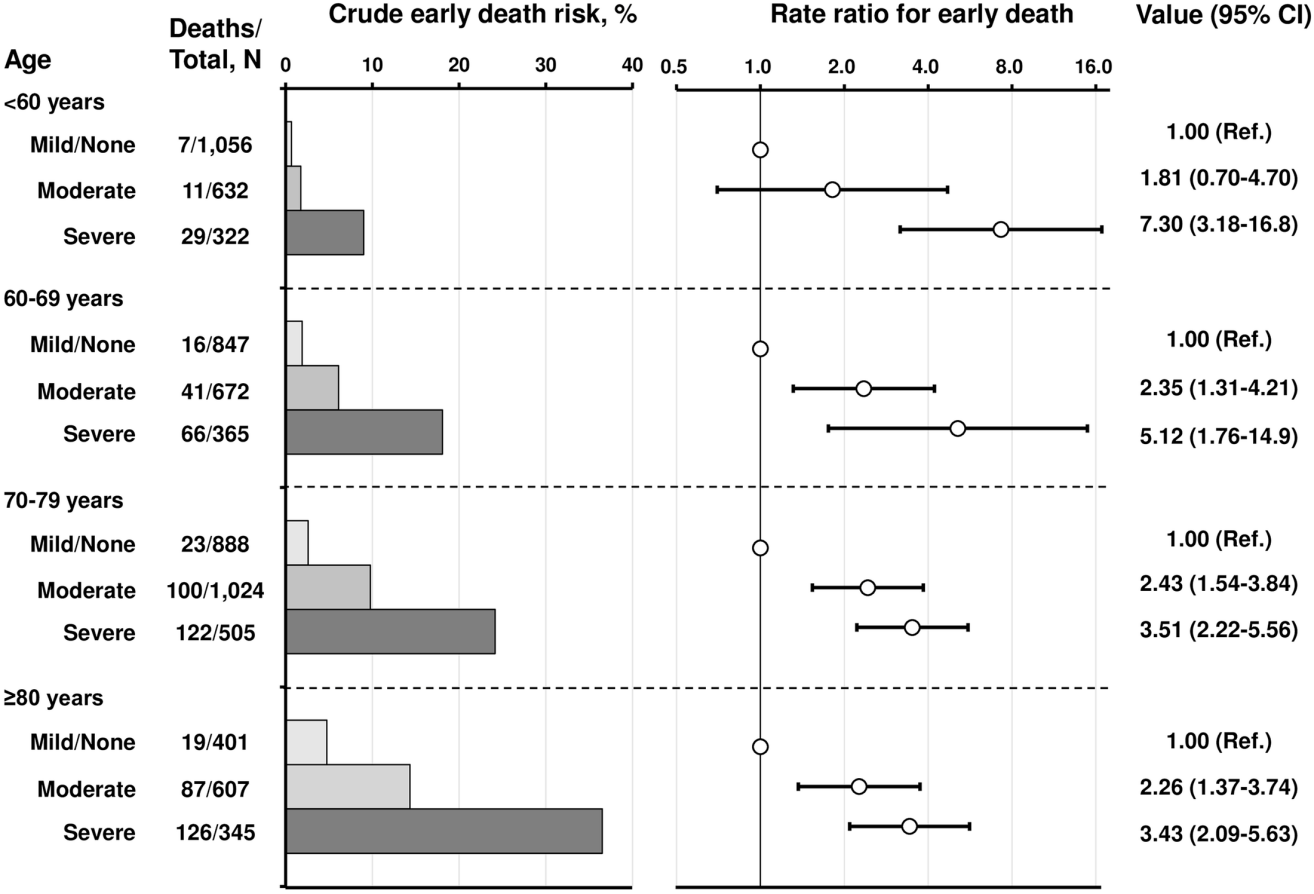
Early death risk and the association with functional status at the start of hemodialysis, accounting for patient age (n = 7,664). Risk ratios for early death were adjusted for the difference in distribution of covariates among patients categorized into 12 subgroups using modified Poission regression models with robust variance. The axis of the rate ratio for early death was represented with a logged scale. Error bars denote 95% CIs. Abbreviation: CI, confidence interval.

The prevalence of moderate to severe functional disability increased with patients’ age (Fig 3) and was particularly high among those aged ≥80 years (severe group, 25%; moderate or severe group, ~70%). The incidence of early death among those in the severe group aged ≥80 years reached as high as 36.5% (Fig 4). The crude risk for early death was higher in the lower FS groups for each age category (Fig 4). The risk ratios for early death among those aged ≥80 years in the moderate and severe groups were 2.26 (95% CI: 1.37–3.74) and 3.43 (95% CI: 2.09–5.63), respectively, compared with the incidence in the mild/none group. This trend persisted regardless of age, indicating that even in the younger population, moderately to severely impaired FS increased the risk of early death (risk ratios of early death were 7.30 in patients aged <60 years, 5.12 in those aged 60–69 years, 3.51 in those aged 70–79 years, and 3.43 in those aged ≥80 years).

Vascular diseases and infections were the most common causes of death across all age categories, and regardless of FS, 50–70% of patients died of vascular diseases or infections (S3 Table). The prevalence of death due to vascular diseases tended to decrease with worsening FS, whereas the opposite trend was observed for deaths due to infections.

## Discussion

Our main findings were as follows. First, even among Japanese patients who have a better prognosis for dialysis, early death was common, especially among elderly patients. Second, impaired FS at initiation of dialysis was significantly associated with early death and death within 1 year after starting dialysis.

The prevalence of early death in Japan is 7.1%, which is in accordance with reports from Western registry data (Canadian Organ Replacement Register [CORR], 5.6%; European Renal Association-European Dialysis and Transplant Association Registry [ERA-EDTA], 6.4%; and US Renal Data System [USRDS], 8.6%) [21–23]. Interestingly, in our study, early death accounted for half of all deaths within 12 months after starting dialysis, which is much higher than that in Western countries (CORR, 36%; ERA-EDTA, 35%; USRDS, 36%) [2]. The reason for a higher incidence of early death in Japanese patients on HD, despite their excellent mid- to long-term survival, may be due to the medical environment in Japan. In Japan, most patients with ESKD start dialysis regardless of how short their expected lifespan may be as long as dialysis is technically feasible. There are several reasons for this: 1) physicians are not exempt from lawsuits for not starting life-prolonging treatments; and 2) no consensus or guidelines for not starting dialysis have been established in Japan. However, given that the main reason for starting dialysis in patients with ESKD is to prolong survival, the policy of dialysis initiation in patients for whom survival is limited may need re-evaluated.

Perhaps the most novel and intriguing finding of our study is that FS at initiation of HD was associated with the incidence of early death within 3 months after initiation of HD. This is the first report focusing on FS at initiation of HD as a predictor for early death. Our study showed that even patients with only moderately impaired FS are at risk of early death, which is relevant for several reasons. First, given the high prevalence of patients on incident dialysis experiencing early death in Japan, identifying those most likely to survive for only a short period after starting dialysis will allow doctors to consider not starting dialysis in these patients. Second, nephrologists tend to attribute impaired FS to uremia, which should be reversed by starting dialysis; however, starting HD may further impair FS and consequently reduce survival [11]. One reason for this discrepancy is that FS impairment or frailty is caused by uremia and non-uremic factors such as aging, inflammation, and co-morbidities, as well as factors associated with dialysis itself, such as hemodynamic instability and catheter use [24]; however our study showed that impaired FS per se is an independent risk for early death after adjusting for several prognostic factors. Mounting evidence suggests that frailty is also an independent risk for mortality [13]. Japanese scoring systems used to assess indications for dialysis consider impaired FS to be a good indication, and common practice in Japan favors starting HD even in those with very poor FS [14].

Regarding patient age, our study uncovered two novel findings: the absolute risks of early death increased with age for all FS categories, and severely impaired FS was a significant risk of early death regardless of age. In our study population, moderately and severely impaired FS were common. Further, the incidence of early death in patients aged ≥80 years with severely impaired FS was extremely high compared to that in patients aged <60 years with severely impaired FS, however, even in younger patients, severely impaired FS confers a great risk of early death, which may be important to consider. Doctors should be particularly prudent in initiating dialysis in frail elderly patients with ESKD since these individuals have a relatively short survival.

Several limitations to the present study warrant mention. First, FS was not an objective index, which may have led to some misclassification. However, even markedly subjective estimates of FS, such as those obtained by asking the surprise question, have been reported as significant predictors of the short-term prognosis of patients on dialysis [25, 26]. We believe that even a subjective estimation tool can predict early death among patients on dialysis. Additionally, we performed a sensitivity analysis to exclude the possibility of misclassification between mild/none and moderate impairment of FS; and proved that the severe group had a significantly higher risk for early death than the non-severe group, suggesting that FS evaluation in our study was valid and useful for predicting mortality. Second, selection bias may exist. The number of patients with FS data was 7,664, accounting for only one-fifth of the entire cohort. This cohort was much older and had a higher early mortality than the overall and excluded cohorts, which may have led to possible overestimation and confounding potential associations. However, we remain confident that the generalizability of the results was sufficiently valid, as the population was large enough to contain a wide range of baseline characteristics. Moreover, representativeness of the study participants is not necessarily required to verify the generalizability of any observed relationship between outcomes and exposures [27]. Finally, the study was observational, preventing us from establishing causal relationships between FS and early death. For example, some medical variables such as the mental status and dialysis adequacy parameters and socio-economic variables were not collected.

In conclusion, early death within 3 months after initiation of dialysis was markedly common among Japanese patients who typically have a good long-term prognosis. Such early deaths represented half of all deaths within 1 year of starting dialysis. Impaired FS at initiation of dialysis was significantly associated with early death, which was irrespective of age at initiation.

## Supporting Information

S1 Table Results of *post-hoc* multiple comparisons with Bonferroni procedure in baseline characteristics among those with three levels of functional disability. (PDF)

S2 Table Multivariable-adjusted risk ratios of each baseline characteristic for early death after initiation of hemodialysis. (PDF)

S3 Table Distribution of Primary Cause of Early Mortality Within 3 months of the Initiation of Hemodialysis in Japan, Stratified by Functional Status and Age. (PDF)
