# Correction: Early Mortality Was Highly and Strongly Associated with Functional Status in Incident Japanese Hemodialysis Patients: A Cohort Study of the Large National Dialysis Registry

**DOI:** 10.1371/journal.pone.0168811

**Published:** 2016-12-19

**Authors:** Masahiko Yazawa, Ryo Kido, Seiji Ohira, Takeshi Hasegawa, Norio Hanafusa, Kunitoshi Iseki, Yoshiharu Tsubakihara, Yugo Shibagaki

There are errors in the Funding section. The correct funding information is as follows: The authors have no support or funding to report.
