# Supplementary material for: Early Mortality Was Highly and Strongly Associated with Functional Status in Incident Japanese Hemodialysis Patients: A Cohort Study of the Large National Dialysis Registry
Source: PLoS One. 2016 Jun 7;11(6):e0156951. doi: 10.1371/journal.pone.0156951 (PMC4896445; doi:10.1371/journal.pone.0156951)
Supplement: S1 Table — (PDF) [file pone.0156951.s001.pdf]

S1 Table. Results of *post-hoc* multiple comparisons with Bonferroni procedure in baseline characteristics among those with three levels of functional disability.

Variables	Compared levels of functional disability		
	Mild / None vs. Moderate	Mild / None vs. Severe	Moderate vs. Severe
Demographic and clinical characteristics			
Age, years	✓	✓	
Sex, female	✓	✓	
Body mass index		✓	
Cause of end-stage kidney disease			
Chronic glomerulonephritis	✓	✓	
Diabetic nephropathy	✓		
Glomerulosclerosis			
Rapid progressive glomerulonephritis		✓	✓
Others		✓	✓
Systolic blood pressure		✓	✓
Co-morbid conditions			
Congestive heart failure	✓	✓	✓

Ischemic heart disease	✓	✓	
Stroke	✓	✓	
Diabetes mellitus	✓		
Malignancy	✓	✓	
Hemiplagia	✓	✓	
Dementia	✓	✓	✓
Liver disease	✓		
Past history of amputation	✓		
Dialysis			
Late referral to nephrologist†		✓	✓
Type of vascular access§			
Arteriovenous fistla	✓	✓	✓
Temporary catheter	✓	✓	✓
Others			
Treatment time		✓	
Laboratory data			
Albumin	✓	✓	✓
Hemoglobin	✓	✓	
Estimated glomerular filtration rate	✓	✓	

C-reaction protein	✓	✓	✓
Calcium‡	✓	✓	✓
Phosphorus			✓

✓: Statistically significant difference was shown between two groups by Mann-Whitney U test for continuous variables or χ^2 test for categorical variables.

†: Late referral was defined as 90 days or less from the first day of visit to the initiation of dialysis.

‡: Albumin-adjusted value was used: calcium+4.0-albumin (if albumin level is less than 4.0 g/dL).
