# Supplementary material for: Early Mortality Was Highly and Strongly Associated with Functional Status in Incident Japanese Hemodialysis Patients: A Cohort Study of the Large National Dialysis Registry
Source: PLoS One. 2016 Jun 7;11(6):e0156951. doi: 10.1371/journal.pone.0156951 (PMC4896445; doi:10.1371/journal.pone.0156951)
Supplement: S2 Table — (PDF) [file pone.0156951.s002.pdf]

S2 Table. Multivariable-adjusted risk ratios of each baseline characteristic for early death after initiation of hemodialysis.

Variables	Rate ratio (95%CI)
Demographic and clinical characteristics	
Age, years (vs <60)	
60 to 69	2.13 (1.57 to 2.90)
70 to 79	2.45 (1.82 to 3.30)
≥80	3.20 (2.34 to 4.38)
Sex, female (vs. male)	0.80 (0.69 to 0.92)
Body mass index, kg/m^2 (vs. 18.5 to 24.9)	
<18.5	1.17 (0.77 to 1.80)
≥25	1.07 (0.68 to 1.69)
Cause of end-stage kidney disease (vs. Chronic glomerulonephritis)	
Diabetic nephropathy	0.88 (0.64 to 1.22)
Glomerulosclerosis	1.24 (0.89 to 1.74)
Rapid progressive glomerulonephritis	1.95 (1.36 to 2.80)
Others	1.82 (1.17 to 2.82)
Systolic blood pressure, mmHg (vs. 120 to 139)	
<100	1.44 (0.92 to 2.27)
100 to 119	1.16 (0.78 to 1.73)
140 to 159	0.91 (0.50 to 1.65)
≥160	0.63 (0.38 to 1.08)
Levels of functional disability (vs. Mild/none)	
Moderate	2.38 (1.80 to 3.10)
Severe	3.93 (2.96 to 5.22)
Co-morbid conditions	
Congestive heart failure, yes (vs. no)	1.19 (0.97 to 1.46)
Ischemic heart disease, yes (vs. no)	1.13 (0.91 to 1.39)
Stroke, yes (vs. no)	0.93 (0.53 to 1.62)
Diabetes mellitus, yes (vs. no)	1.08 (0.80 to 1.45)
Malignancy, yes (vs. no)	1.14 (0.76 to 1.72)
Hemiplegia, yes (vs. no)	0.86 (0.51 to 1.43)
Dementia, yes (vs. no)	1.13 (0.94 to 1.36)
Liver disease, yes (vs. no)	1.28 (0.99 to 1.68)
Past history of amputation, yes (vs. no)	1.02 (0.58 to 1.77)

Dialysis	
Late referral to nephrologist†, yes (vs. no)	1.28 (0.91 to 1.81)
Type of vascular access (vs. Arteriovenous fistula)	
Temporary catheter	1.98 (1.03 to 3.82)
Others	1.91 (1.47 to 2.49)
Treatment time, hours, <4.0 (vs. ≥4.0)	1.67 (0.68 to 4.12)
Laboratory data	
Albumin, g/dL (vs. ≥4.0)	
<3.0	2.30 (1.28 to 4.12)
3.0 to 3.4	1.61 (0.81 to 3.19)
3.5 to 3.9	1.44 (0.73 to 2.83)
Hemoglobin, g/dL (vs. ≥10.0)	
<8.0	0.96 (0.69 to 1.33)
8.0 to 9.9	0.97 (0.63 to 1.33)
Estimated glomerular filtration rate, ml/min/1.73 m^2§ (vs. ≥6.5)	
3.5 to 6.4	0.75 (0.63 to 0.90)
<3.5	1.13 (0.33 to 3.83)
C-reaction protein, mg/dL, >0.3 (vs. ≤0.3)	1.83 (1.10 to 3.05)
Calcium, mg/dL‡ (vs. 8.4 to 9.9)	
<8.4	1.12 (0.32 to 4.00)
≥10.0	1.42 (0.88 to 2.29)
Phosphorus, mg/dL (vs. 4.0 to 5.9)	
<4.0	1.05 (0.52 to 2.11)
6.0 to 7.9	1.02 (0.80 to 1.31)
≥8.0	1.13 (0.79 to 1.62)

†: Late referral was defined as 90 days or less from the first day of visit to the initiation of dialysis.

§: Estimated glomerular filtration rate was calculated from the formula for Japanese as follows: 194 × (serum creatinine, mg/dL)$^{-1.094}$ 1.094 creatini$^{-0.287}$, and 0.739 if female

‡: Albumin-adjusted value was used: calcium + 4.0 – albumin (if albumin level <4.0 g/dL)
