# Supplementary material for: Early Mortality Was Highly and Strongly Associated with Functional Status in Incident Japanese Hemodialysis Patients: A Cohort Study of the Large National Dialysis Registry
Source: PLoS One. 2016 Jun 7;11(6):e0156951. doi: 10.1371/journal.pone.0156951 (PMC4896445; doi:10.1371/journal.pone.0156951)
Supplement: S3 Table — (PDF) [file pone.0156951.s003.pdf]

S3 Table. Distribution of Primary Cause of Early Mortality Within 3 months of the Initiation of Hemodialysis in Japan, Stratified by Functional Status and Age. (n = 7,664).

	No. of death / No. of patient, n (%)		Primary Cause of Death			
			Cancer, n (%)	Vascular disease, n (%)	Infection, n (%)	Others, n (%)
Study subjects						
Total	647 / 7,664	(8.4%)	61 (9.4%)	207 (32.0%)	190 (29.4%)	189 (29.2%)
Age <60, years						
Levels of functional disability						
Mild / None	7 / 1,056	(0.7%)	2 (28.6%)	3 (42.9%)	1 (14.3%)	1 (14.3%)
Moderate	11 / 632	(1.7%)	2 (18.2%)	4 (36.4%)	3 (27.3%)	2 (18.2%)
Severe	29 / 322	(9.0%)	6 (20.7%)	9 (31.0%)	7 (24.1%)	7 (24.1%)
Age 60 to 69, years						
Levels of functional disability						
Mild / None	16 / 847	(1.9%)	1 (6.3%)	8 (50.0%)	4 (25.0%)	3 (18.8%)
Moderate	41 / 672	(6.1%)	5 (12.2%)	9 (22.0%)	14 (34.2%)	13 (31.7%)
Severe	66 / 365	(18.1%)	7 (10.6%)	19 (28.8%)	22 (33.3%)	18 (27.3%)
Age 70 to 79, years						
Levels of functional disability						
Mild / None	23 / 888	(2.6%)	2 (8.7%)	6 (26.1%)	4 (17.4%)	11 (47.8%)
Moderate	100 / 1,024	(9.8%)	12 (12.0%)	32 (32.0%)	22 (22.0%)	34 (34.0%)
Severe	122 / 505	(24.2%)	10 (8.2%)	45 (36.9%)	40 (32.8%)	27 (22.1%)
Age ≥80, years						

Levels of functional disability						
Mild / None	19 / 401	(4.7%)	1 (5.3%)	7 (36.8%)	5 (26.3%)	6 (31.6%)
Moderate	87 / 607	(14.3%)	7 (8.1%)	26 (29.9%)	25 (28.7%)	29 (33.3%)
Severe	126 / 345	(36.5%)	6 (4.8%)	39 (31.0%)	43 (34.1%)	38 (30.2%)

Note: Values for categorical variables are given as numbers (percentage).
